# Fine tuning of the net charge alternation of polyzwitterion surfaced lipid nanoparticles to enhance cellular uptake and membrane fusion potential

**DOI:** 10.1080/14686996.2024.2338785

**Published:** 2024-04-10

**Authors:** Keitaro Homma, Yutaka Miura, Motoaki Kobayashi, Wanphiwat Chintrakulchai, Masahiro Toyoda, Koichi Ogi, Junya Michinishi, Tomoyuki Ohtake, Yuto Honda, Takahiro Nomoto, Hiroyasu Takemoto, Nobuhiro Nishiyama

**Affiliations:** a Laboratory for Chemistry and Life Science, Institute of Innovative Research, Tokyo Institute of Technology, Kanagawa, Japan; b Department of Life Science and Technology, School of Life Science and Technology, Tokyo Institute of Technology, Kanagawa, Japan; c I&S Department, Corporate R&D division, NOF CORPORATION, Kanagawa, Japan; d Innovation Center of Nanomedicine (iCONM), Kawasaki Institute of Industrial Promotion, Kanagawa, Japan; e Department of Life Sciences, Graduate School of Arts and Sciences, The University of Tokyo, Tokyo, Japan; f Medical Chemistry, Graduate School of Medical Science, Kyoto Prefectural University of Medicine, Kyoto, Japan

**Keywords:** Lipid nanoparticles, siRNA, polycarboxybetaine, membrane fusion, pH-responsiveness

## Abstract

Lipid nanoparticles (LNPs) coated with functional and biocompatible polymers have been widely used as carriers to deliver oligonucleotide and messenger RNA therapeutics to treat diseases. Poly(ethylene glycol) (PEG) is a representative material used for the surface coating, but the PEG surface-coated LNPs often have reduced cellular uptake efficiency and pharmacological activity. Here, we demonstrate the effect of pH-responsive ethylenediamine-based polycarboxybetaines with different molecular weights as an alternative structural component to PEG for the coating of LNPs. We found that appropriate tuning of the molecular weight around polycarboxybetaine-modified LNP, which incorporated small interfering RNA, could enhance the cellular uptake and membrane fusion potential in cancerous pH condition, thereby facilitating the gene silencing effect. This study demonstrates the importance of the design and molecular length of polymers on the LNP surface to provide effective drug delivery to cancer cells.

## IMPACT STATEMENT

The study presents the unique characteristics of small interfering RNA (siRNA)-loaded lipid nanoparticles (LNPs) with different lengths of PGlu(DET-Car), revealing the length of PGlu(DET-Car) critically affects the formation of a stable LNP, the cellular uptake, membrane fusion, and gene silencing abilities.

## GRAPHICAL ABSTRACT

**Figure:**
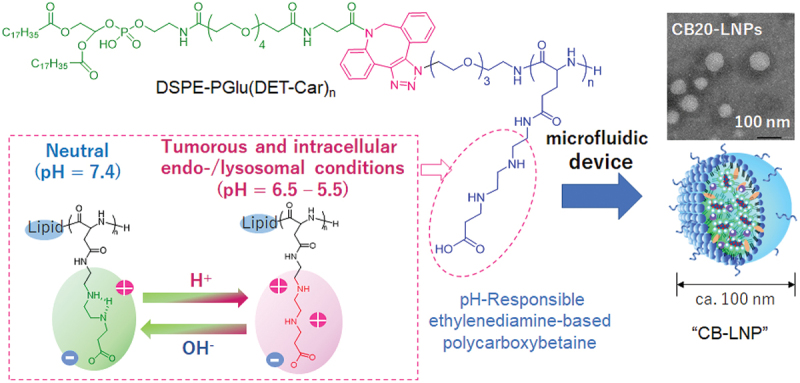

## 1. Introduction

Engineering of lipid nanoparticles (LNPs) has given rise to substantial breakthroughs toward the utilization of nanomedicines to deliver small interfering RNA (siRNA) and messenger RNA (mRNA) [1–3]. In particular, the practical utility of systemically injectable LNPs for transthyretin-related hereditary amyloidosis, *i.e*. Onpattro [4], and intramuscularly injectable LNPs for SARS-CoV-2 vaccine, *i.e*. Comirnaty [5] have recently been demonstrated as effective therapeutics. Nevertheless, LNPs have not achieved translational success in tumor therapy through systemic injections. Because LNPs are nano-sized self-assembled from many components such as lipids [6–8], stabilizers [9,10], and modifiers [11,12], and they require particularized tuning for their formation and to ensure their physical and chemical properties as cancer applicable nanomedicines [13,14]. Especially, the RNA incorporated in the LNPs must be stable during blood circulation, but after arrival at the targeted site, the surface of the LNPs should interact appropriately with the cell membrane around tumor sites. This interactive property is essential to facilitate the cellular uptake, endosomal escape, and therapeutic outcomes. Although poly(ethylene glycol) (PEG) is a well-known surface-modifying material and is utilized to provide the antifouling property to the LNP surface, the PEGylated LNP system is often confronted with inefficient drug delivery due to the hindrance of the above interaction between the cell membrane and PEG-modified LNPs [15,16]. Therefore, to develop LNPs for effective cancer therapy, it is crucial to design the surface of drug incorporated LNPs considering the potentials of the membrane interactions.

**Figure 1.**
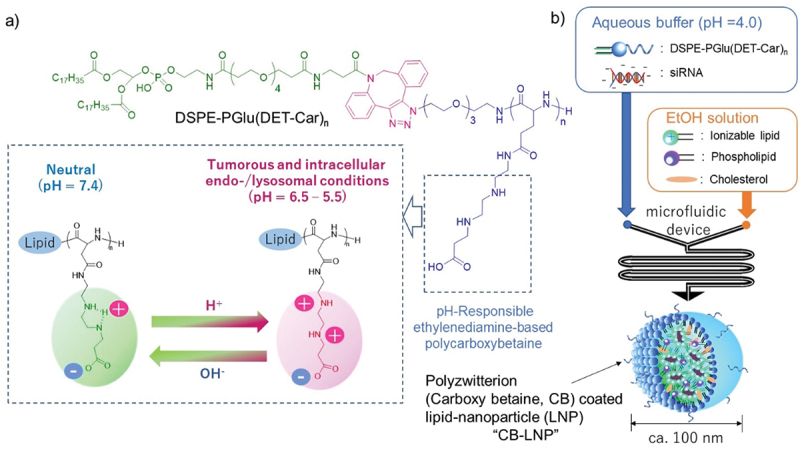
Preparation method of polyzwitterion coated lipid-nanoparticle (LNP) (CB-LNP). (a) Chemical structure and the protonation functionality. (b) Summary of LNP preparations using a microfluidic device.

Regarding the surface materials, we previously designed and synthesized poly(*N*-{*N*’-[*N*”-(2-carboxyethyl)-2-aminoethyl]-2-aminoethyl}glutamide), [PGlu(DET-Car)], to introduce it onto the surface of nanoparticles [17–20]. In a neutral environment (pH 7.4), the ethylenediamine moiety on the PGlu(DET-Car) side chain is a monovalent cation in the gauche structure and becomes electrically neutral by interacting with the monovalent anion of the carboxyl group. On the other hand, under cancerous acidic pH (pH = 6.5) conditions, the ethylenediamine moiety becomes an anti-conformer of the divalent cation. Therefore, PGlu(DET-Car)-coated nanoparticles demonstrate antifouling behavior in neutral blood conditions, and the surface of the nanoparticles shifts to electrically positive to facilitate the membrane interactions at the tumor site (Figure 1(a)). However, these performances in previous studies were revealed using the limited value of degree of polymerization (DP) in the PGlu(DET-Car) sequence, and thus the molecular design in the developed PGlu(DET-Car)-coated nanoparticles has not been fully optimized in terms of engineering aspects. The relationship among the amount of polybetaine side chains, electrostatic interaction on cell membrane, and related drug delivery functions remains controversial.

To investigate the LNP functions with regards to the cell membrane, a membrane fusion assay is a powerful tool [21,22]. Membrane fusion was originally determined as essential bioprocesses such as neurotransmission [23,24], viral infection [25,26], and exocytosis [27], but the recent development of membrane fusion phenomena and the methodology involved are useful to understand drug delivery efficiency which can feedback to the design of LNPs [28,29]. Thus, in this study, we aimed to clarify the effects of the DP value in the PGlu(DET-Car) segment on the LNPs to maximize the ability to detect the narrow pH window under various physiological conditions such as blood stream, tumor microenvironment and endo-/lysosomal compartment. We prepared siRNA-loaded LNPs (siRNA-LNPs) with different lengths of PGlu(DET-Car), and evaluated their *in vitro* activity and membrane fusion potential to confirm the impact on the efficacy of drug delivery. Our results demonstrate that the length of PGlu(DET-Car) critically affects the formation of a stable siRNA-LNP, cellular uptakes, membrane fusions, and gene silencing abilities. These results indicate the advantage of utilizing PGlu(DET-Car) as next-generation structural components for drug delivery system (DDS) carriers.

## 2. Materials and methods

### 2.1. Reagents

*N*, *N*-dimethylformamide (DMF), dichloromethane (DCM), diethyl ether, 5 M HCl, diethyl ether, methanol, ethanol, tetrahydrofuran (THF), acetonitrile, sodium chloride, 4-(2-hydroxyethyl)-1-piperazineethanesulfonic acid (HEPES), 2-morpholinoethanesulfonic acid (MES), and tris(hydroxymethyl)aminomethane hydrochloride salt (Tris-HCl) were purchased from FUJIFILM Wako Pure Chemical Co. Ltd. (Osaka, Japan). Diethylenetriamine, *tert*-butylacrylate, 2-hydroxyprydine, and sodium acetate were purchased from Tokyo Chemical Industry Co., Ltd. (Tokyo, Japan). Cholesterol and Dulbecco’s Phosphate-Buffered Saline were purchased from Nacalai Tasque, Inc. (Kyoto, Japan). Acetic acid was purchased from Kanto Chemical Industry Co., Ltd. (Tokyo, Japan). γ-benzyl-L-glutamate *N*-carboxy anhydride (BLG-NCA) was purchased from Chuo Kaseihin Co., Inc. (Tokyo, Japan). 1,2-dioleoyl-3-dimethylammonium-propane (DODAP), 1,2-dioleoyl-sn-glycero-3-phosphoethanolamine (DOPE), 1,2-dioleoyl-*sn*-glycero-3-phospho-L-serine (sodium salt) (DOPS), and 1,2-dioleoyl-*sn*-glycero-3-phosphoethanolamine-*N*-(lissamine rhodamine B sulfonyl) (ammonium salt) (Rhodamine-PE) were purchased from Avanti Polar Lipids, Inc (Alabaster, Alabama, U.S.A.). Polyethyleneglycol-1,2-dioleoyl-*sn*-glycero-3-phosphoethanolamine (PEG5k-DSPE) and 1,2-dioleoyl-*sn*-glycero-3-phosphocholine (DOPC) were purchased from NOF Co., Ltd. (Tokyo, Japan). 1,2-Distearoyl-*sn*-glycero-3-phosphoethanolamine-*N*-tetra(polyethylene glycol)-dibenzocyclooctyne (DSPE-PEG_4_-DBCO) was purchased from Broadpharm (San Diego, California, U.S.A.). 1-amino-11-azido-3,6,9-trioxaundecane, iT™ X-100, penicillin/streptomycin, trypsin/ethylenediamine tetra-acetic acid (EDTA), Quant-RiboGreen® RiboGreen RNA Assay Kit, RiboGreen RNA Reagent, McCoy’s 5A (modified) medium, RiboGreen®, Triton™ Red DND-99, it™ 33342, and Trypan blue were purchased from Sigma Aldrich (St. Louis, Missouri, U.S.A.). Small interfering RNA (siRNA) against luciferase (siLuc, sense: 5’-GAu UAu GuC CGG uuA uGu AUU AdTdT-3’; antisense: 5’-UAC AuA ACC GGA CAu AAu CUU GdTdT-3’, 2’-Ome-modified nucleotides are in lowercase.) were purchased from Hokkaido System Science Co., Ltd. (Hokkaido, Japan). Alexa 647-labeled siRNA (Alexa 647-siGL3, sense: 5’-CUU ACG CUG AGU ACU UCG AdTdT-3’; antisense: 5’-Alexa 647-UCG AAG UAC UCA GCG UAA GdTdT-3’) was prepared using Gene Design, Inc. (Osaka, Japan).

### 2.2. Synthesis of PBLG

Poly(γ-benzyl-L-glutamate) (PBLG) was synthesized by ring-opening polymerization of γ-benzyl-L-glutamate *N*-carboxy anhydride (BLG-NCA) with 11-azido-3,6,9-trioxaundecan-1-amine as an initiator. BLG-NCA (1.0 g, 3.73 mmol) was dissolved in 20 mL of a mixture of DMF and DCM (1/4, *v*/*v*) under Ar. Then, 11.6 mg (0.053 mmol) of the initiator was added to the mixture and stirred at room temperature for two days. The reaction solution was added to an excess amount of diethylether (200 mL), and the precipitate was dried in vacuum to obtain a white powder (0.63 g, 0.047 mmol, 80% yield). PBLG was characterized by ^1^H NMR spectroscopy (AVANCE III 400, Bruker, Billerica, MA). ^1^H NMR (DMSO-*d*6): δ(ppm) = 8.08–8.62 (br,42 H, NH-(COCHN*H*)-H), 7.07–7.49 (br, 525 H, –CH_2_C_6_*H*_5_), 4.90–5.18 (br, 187 H, –C*H*_2_C_6_H_5_), 3.70–4.26 (br, 86 H, -NH-(COC*H*NH)-H), 3.39–3.60 (m, 16 H, N_3_C*H*_2_C*H*_2_-(OC*H*_2_C*H*_2_)_3_-NH-), 1.78–2.32 (br, 142 H, -CHC*H*_2_C*H*_2_COO).

### 2.3. *Synthesis of DET-Car-*t*Bu*

DET-Car-*t*Bu was synthesized using the Michael reaction of *tert*-butylacrylate with diethylenetriamine. *Tert*-butylacrylate (23 g, 162 mmol) was added to an excess of diethylenetriamine (100 g, 971 mmol) and stirred at room temperature for 2 h. DCM (200 mL) was added to reacted solution. The DCM solution washed with saturated NaCl_(aq)_ (300 mL × 2). The DCM was then removed using a rotary evaporator. MeOH (100 mL) and 1 M HCl_(aq)_ was added to the remaining oily substance to ionize the product. White crystals were obtained after recrystallization with diethylether (10 mL) at −20°C, and the corrected white crystal was dried under vacuum to obtain a white powder (19.5 g, 84 mmol, 52% yield). DET-Car-*t*Bu was characterized by ^1^H NMR spectroscopy (AVANCE III 400). 1 H-NMR (D_2_O): δ(ppm) = 3.31–3.64 (m, 10 H, NH_2_C*H*_2_C*H*_2_NHC*H*_2_C*H*_2_NHC*H*_2_CH_2_), 2.77–2.85 (t, 2 H, -CH_2_C*H*_2_COO), 1.44–1.52 (s, 9 H, -C(C*H*_3_)_3_).

### 2.4. Synthesis of PGlu(DET-Car)

PGlu(DET-Car) was synthesized with the aminolysis reaction of PBLG with DET-Car-*t* Bu. Before the reaction, DET-Car-*t* Bu was neutralized with 1 M NaOH_(aq)_. PBLG (100 mg, 7.48 µmol, 0.449 mmol of the benzyl group) and 2-hydroxypyridine (215 mg, 2.25 mmol, 5 eq. to the benzyl group in PBLG) was dissolved in 5 mL of THF, followed by the addition of DET-Car-*t* Bu (2.29 g, 6.74 mmol, 15 eq. to the benzyl group in PBLG), The mixtures were stirred for 2 days at room temperature prior to dialysis (molecular weight cut-off: 3.5k Da) against HCl (1 eq. of the reacting amine groups), followed by further two dialysis steps against water. Then, 1 M HCl (5 mL) was added to the mixture to deprotect *t*Bu group, followed by stirring at 40°C for 2 days. After dialysis against deionized water (500 mL × 3), the final solution was lyophilized to obtain a white powder (97.5 mg, 5.61 µmol, 75% yield). PGlu(DET-Car) was characterized by ^1^H NMR spectroscopy (AVANCE III 400). ^1^H-NMR (D_2_O): δ(ppm) = 4.26–4.49 (br, 1 H, NH-(COC*H*NH)-H), 3.67–3.75 (m, N_3_-C*H*_2_C*H*_2_-(OC*H*_2_C*H*_2_)-NH), 3.27–3.66 (m, 10 H, NH-C*H*_2_C*H*_2_-NH-C*H*_2_C*H*_2_-NHC*H*_2_), 2.86–2.95 (m, 2 H, C*H*_2_CH_2_COOH), 2.32–2.56 (m, 2 H, -CH_2_C*H*_2_CONH), 1.91–2.25 (m, 2 H, -C*H*_2_CH_2_CONH).

### 2.5. Synthesis of DSPE-PGlu(DET-Car) (CB-DSPE)

DSPE-PGlu(DET-Car) was synthesized by the click reaction of PGlu(DET-Car) with DSPE-PEG_4_-DBCO. PGlu(DET-Car) (30 mg, 1.73 µmol) was dissolved in deionized water (1.5 mL) and DSPE-PEG_4_-DBCO (5 mg, 3.90 µmol, 2.3 eq) was DCM (1.5 mL), and the solutions were mixed. The mixtures were stirred for two days at room temperature. The reaction solution was added to an excess amount of acetonitrile (60 mL) and stirred for 2 days at room temperature. The collected precipitate was dissolved in deionized water and lyophilized to obtain a white powder (25 mg, 1.34 µmol, 75% yield). DSPE-PGlu(DET-Car) was characterized by ^1^H NMR spectroscopy (AVANCE III 400). ^1^H-NMR (D_2_O):δ(ppm) = 4.16–4.49 (br, 1 H, NH-(COC*H*NH)-H), 3.68–3.86 (m, N_3_-C*H*_2_C*H*_2_-(OC*H*_2_C*H*_2_)-NH), 3.00–3.66 (m, 10 H, NH-C*H*_2_C*H*_2_-NH-C*H*_2_C*H*_2_-NHC*H*_2_), 2.53–2.69 (m, 2 H, CH_2_C*H*_2_COOH), 2.26–2.49 (m, 2 H, -CH_2_C*H*_2_CONH), 1.91–2.23 (m, 2 H, -C*H*_2_CH_2_CONH), 1.14–1.39 (m, -C*H*_2_-), 0.68–0.95 (m, -C*H*_3_).

### 2.6. Preparation and characterization of LNPs

To prepare pH-responsive polycarboxybetaine (CBx; note that x refers to the degree of polymerization) 20-, 70-, and CB110-LNPs, siRNA/CB20-DSPE, siRNA/CB70-DSPE or siRNA/CB110-DSPE aqueous solution (siRNA = 5.3 µM, acetate buffer = 25 mM, pH 4.0) was mixed with lipid ethanol solution (DODAP/DOPE/cholesterol = 50/10/40 molar ratio) through NanoAssembler Ignite^TM^ (Precision NanoSystems, Vancouver, Canada; aqueous solution/ethanol solution, 3/1). To prepare CB20-LNPs and PEG-LNPs with different N/P ratio, we used different siRNA concentrations accordingly (note that the N stands for the molar amount of DODAP, and the P represents the molar amount of the bases in siRNA). The obtained LNPs were purified by dialysis (Spectra/Pro 2 Membrane: molecular weight cut-off, 12k−14k Da) with 10 mM HEPES buffer (pH 7.4) [30]. Size and ζ potential of the obtained LNPs were measured by a Zetasizer Nano ZS Instrument (Malvern Instruments Ltd., Worcestershire, UK). For the PEG-LNPs as control, lipid ethanol solutions containing PEG-DSPE were used as an alternative. The siRNA encapsulation efficiency was determined using the RiboGreen assay [31]. First, total siRNA was estimated by Quant-iT™ RiboGreen® RNA reagent (Thermo Fisher Scientific, Waltham, Massachusetts, U.S.A.). The LNP samples were diluted with HEPES buffer (pH 7.4, 10 mM) containing 0.4% (*v*/*v*), and then mixed with Triton X-100 and dextran sulfate (80 μg/mL; Merck Millipore, Burlington, Massachusetts, U.S.A.). Separably, the LNP solution which diluted with same HEPES buffer was mixed with RiboGreen® RNA reagent for free siRNA analysis. The fluorescence of total and free siRNA was measured using with a microplate reader (Spark, Tecan Group Ltd., Männedorf, Switzerland) (excitation wavelength = 487 nm, emission wavelength = 537 nm). Separate calibration curves were prepared for each solution to consider the effects of Triton X-100 and dextran sulfate on fluorescence intensities. Encapsulation efficiency (%) was determined by the following equation.

$$\;\;\;\;\;\;\;\;\;\;\;\;\;\;\;\;\;\;\;\;\;\;\;\;\;\;\;\;\;\;\;\;\;\;\;\;\;\;\;\;\;\;\;\;\;\;\;\;\;\;\;\;\;\;\;\;\;\;\;\;\;\;\;\;\;\;\;\;\;\;\;\;\;\;\mathrm{Encapsulation}\;\;\mathrm{rate}\left(\%\right)\;\;=\;\left(\left[\mathrm{total}\;\;\mathrm{siRNA}-\mathrm{free}\;\;\mathrm{siRNA}\right]\;/\mathrm{total}\;\;\mathrm{siRNA}\right)\;\times \;100\%$$

### 2.7. Membrane fusion potential

Biomembrane mimetic lipid mixture containing rhodamine-PE (lipid composition; DOPC/DOPE/DOPS/Chol/Rhodamine-PE = 45/15/15/20/10) was dissolved in chloroform. Lipid films were formed by using a rotary evaporator, and rhodamine-labeled liposomes were prepared by hydration with 10 mM HEPES buffer [32,33]. Each LNP and rhodamine-labeled liposomes were mixed (37°C, 1 h) in a 10/1 molar ratio, and then incubated in buffers at different pH levels (pH 5.5, 6.0, and 6.5 in 10 mM MES with 150 mM NaCl; pH 7.0 and 7.5 in HEPES with 150 mM NaCl; pH 8.0 and 8.5 in 10 mM Tris-HCl with 150 mM NaCl). At 1 h after incubation, the fluorescence intensity (I) was measured using a microplate reader (Spark, Tecan Group Ltd, excitation: 520 nm, emission: 590 nm). The fluorescence intensity of rhodamine-labeled liposomes diluted by 0.1% Triton^TM^ X-100 was defined as the maximum membrane fusion potential. The membrane fusion potential was calculated by the following equation:

$$\mathrm{Membrane}\;\mathrm{fusion}\;\mathrm{potential}=\frac{I_{\mathrm{Liposomes}+\mathrm{LNPs}}-I_{\mathrm{Liposomes}}}{I_{\mathrm{Liposomes}+\mathrm{Triton}}-I_{\mathrm{Liposomes}}}$$

where *I*_Liposomes + LNPs_ refers to the fluorescent intensity of liposomes after the addition of LNPs. *I*_Liposomes_ refers to the fluorescent intensity of liposomes diluted with buffers at different pHs. *I*_Liposomes+Triton_ refers to the fluorescent intensity of liposomes diluted with 0.1% Triton^TM^ X-100.

### 2.8. Endosomal escape

SK-OV-3-luc (5.0 × 10^4^ cells) were seeded on 24-well plates (Iwaki, Tokyo, Japan) and cultured in McCoy’s 5A (modified) medium containing 10% fetal bovine serum (FBS). The cell medium was then replaced with new Optimem containing 250 µM calcein and siLuc encapsulating LNPs. After incubation for 2 h, the medium was washed with D-PBS(−) twice and the nuclei were stained by using Hoechst 33,342 (Dojindo, Kumamoto, Japan). Chloroquine was also used as positive control for endosome escape analysis. The endosomal escape was evaluated by BZ-X800 (KEYENCE, Tokyo, Japan) and quantified using BZ-X800 Analyzer (ver. 1.1.24).

### 2.9. Confocal laser scanning microscopy observation of the incorporation of LNPs

SK-OV-3-luc cells were seeded (1.0 × 10^5^ cells/well) on 35 mm glass base dishes (Iwaki, Tokyo, Japan) and cultured in McCoy’s 5A (Modified) medium containing 10% fetal bovine serum (FBS). The cell medium was then replaced with new Optimem containing Alexa Flour 647-siGL3 encapsulating LNPs. After incubation for 72 h, the nuclei and lysosomes were stained with Hoechst 33,342 (Dojindo, Kumamoto, Japan) and LysoTracker Red DND-99 (Thermo Fisher Scientific, Inc. Waltham, MA, U.S.A.), respectively. LSM710 (Carl Zeiss, Oberkochen, Germany) equipped with a Plan-Apochromat 40× oil immersion objective lens (aperture: 1.4) was used for each confocal laser scanning microscopy (CLSM) measurement. The excitation wavelengths were 405 nm for Hoechst 33,342, 488 nm for LysoTracker Red DND-99, and 633 nm for Alexa Flour 647-siGL3. For quantitative analysis of cellular uptake study, the regions-of-interest (ROIs) were selected manually to identify each single cell (*n* = 30). The fluorescence signals (633 nm) detected from ROIs were quantified using ZEN Microscopy software (ver. 6.2.0.500).

### 2.10. Cell viability assay

SK-OV-3-luc cells were seeded on 96-well plates (Iwaki, Tokyo, Japan, 5.0 × 10^3^ cells/well) and incubated in McCoy’s 5A (Modified) medium containing 10% FBS. After 72 h of incubation, LNPs were removed, and all well plates were washed with phosphate-buffered saline. Then, Optimem containing CCK-8 assay reagent was used for the analysis. (BIO-RAD, Hercules, California, U.S.A.). Cell viabilities were calculated using the following equation:

$$\mathrm{Viability}=\frac{A_{\mathrm{Sample}}-A_{\mathrm{Blank}}}{A_{\mathrm{Control}}-A_{\mathrm{Blank}}}$$

where *A*_Sample_ refers to the absorbance of the well of samples-treated cells. *A*_Control_ refers to the absorbance of the well of untreated cells. *A*_Blank_ refers to the absorbance of the well of medium without cells.

### 2.11. In vitro gene silencing

SK-OV-3-luc cells were treated with the same conditions described in section 2.6. *Cell viability assay*. The cells were incubated with pH-adjusted Optimem containing siLuc-incorporated LNPs. After incubation for 72 h, the cell viability was again confirmed with the method described in section 2.6. The passive lysate buffer (Promega Co.) (50 µL), cell lysate (20 µL), and luciferin substrate (100 µL) were added into each well. The degree of *in vitro* gene viabilities was measured using a luciferase assay system kit (Promega Co.) and a GloMax 96 Microplate Luminometer (Promega Co.) Gene silencing efficiency was calculated using the following equation:

$$\mathrm{Gene}\;\mathrm{silencing}\;\mathrm{efficiency}=\frac{I_{\mathrm{Sample}}/V_{\mathrm{Sample}}}{I_{\mathrm{Control}}/V_{\mathrm{Control}}}$$

where *I*_Sample_ and *V*_Sample_ refer to the luminescent intensity and the viability of the well of treated cells, respectively. *I*_Control_ and *V*_Control_ refer to the luminescent intensity and the viability of the well of untreated cells, respectively.

### 2.12. Statistical analysis

All statistical data are expressed as mean ± standard deviation. The data were analyzed by Student’s *t*-test to determine the statistical significance between the two mean values. Values with *p* < 0.05 were considered statistically significant.

## 3. Results

### 3.1. Preparation and characterization of LNPs

**Figure 2.**
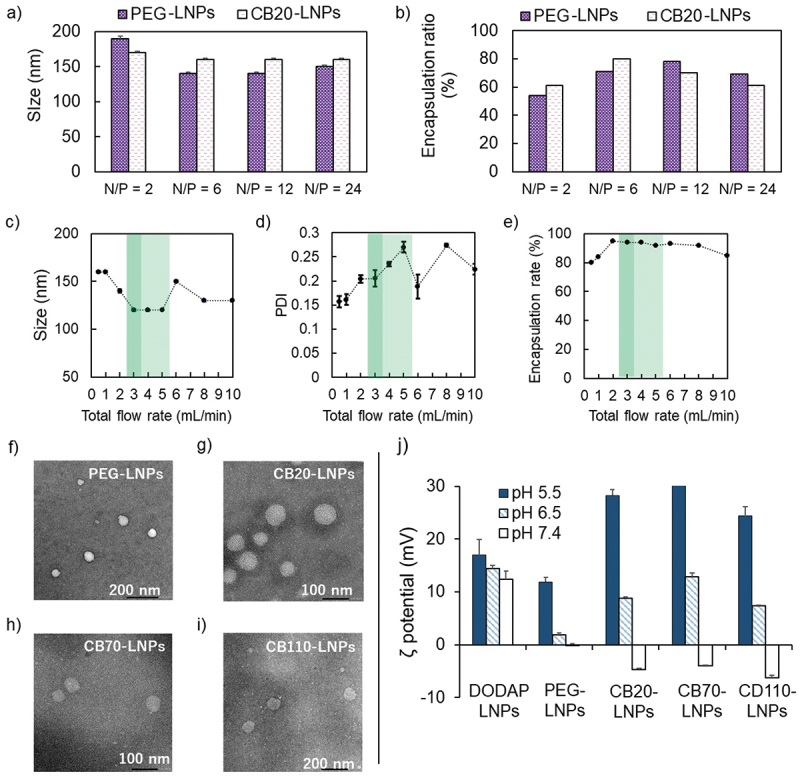
Physicochemical characteristics of lipid nanoparticles (LNPs). (a,b) Initial LNPs formulated at different N/P ratios were prepared by microfluidic device with 0.5 mL/min total flow rate. (a) Size, (b) encapsulation ratio of siRNA. Data are expressed as mean ± standard deviation (SD) (*n* = 3). (c,e) Effect of total flow rate for preparation of LNPs. (c) Size, (d) polydispersity index (PDI), (e) encapsulation rate. (f,i) Transmission electron microscopy images. (j) Relationship between pH and ζ potential of LNPs (*n* = 3).

Differ from our previous report [19], we used a microfluidic device for the preparation of LNPs (Figure 1(b)). The microfluidic device, NanoAssembler Ignite^TM^, is well-known and used for clinically transrational engineering for the preparation of LNPs [34,35] and this method is capable of supplying LNPs with different characteristics such as particle size and polydispersity index (PDI), by tuning their total flow rate during the mixing process [30]. First, we prepared PEG-LNPs and carboxy betaine coated LNPs (CBx-LNPs) using CB-modified lipid (Figure 1) at varying N/P {N/P = [amino groups (N) in the DODAP]/[phosphate groups (P) in the siRNA]} ratios. Note that x represented after CB indicates the degree of polymerization in pH-responsive polycarboxybetaine. The PEG-LNPs and CB20-LNPs with N/P = 2 showed relatively large diameters, but they became almost constant at N/P ratios from 6 to 24 (Figure 2(a)). Encapsulation ratio of siRNA showed the increasing tendency, and the maximum value was obtained when N/P = 6 (Figure 2(b)). Thus, we used this N/P ratio for further preparation of our LNPs. Next, we adjusted the value of total flow rate from 0.5 to 10 mL/min, and we found that the smallest particle size obtained was in the range of 3–5 mL/min (Figure 2(c)). Additionally, both the narrow PDI and high encapsulation efficiency of siRNA were achieved when the total flow rate was 3 mL/min (Figure 2(d,e)). Thus, we defined the above condition as an optimum condition to prepare our CBx-LNPs. Various LNPs were then prepared using the same condition and their physical properties were evaluated and summarized in Table 1. Furthermore, electron microscopy imaging showed that the obtained PEG-, CB20-, CB70-, and CB110-LNPs were almost spherical in shape (Figure 2(f–i)). Based on the TEM images, the quantification of LNP diameter was a little smaller than that of dynamic light scattering data, but they showed similar tendency (Table 1).

**Table 1.** Composition and characteristics of lipid nanoparticles (LNPs).

LNPs	Lipid composition (mol%)	Size (nm)^a^	Size (nm)^b^	PDI^a^	Encapsulation rate^c^
DODAP-LNPs	DODAP/DOPE/Chol = 50/10/40	104 ± 1	N.D.	0.19 ± 0.01	100%
PEG-LNPs	DODAP/DOPE/Chol/PEG-DSPE = 50/10/40/1	96 ± 1	88 ± 2	0.20 ± 0.01	87%
CB20-LNPs	DODAP/DOPE/Chol/CB20-DSPE = 50/10/40/1	121 ± 1	103 ± 3	0.18 ± 0.01	94%
CB70-LNPs	DODAP/DOPE/Chol/CB70-DSPE = 50/10/40/1	114 ± 0.8	103 ± 3	0.24 ± 0.01	60%
CB110-LNPs	DODAP/DOPE/Chol/CB110-DSPE = 50/10/40/1	127 ± 1	122 ± 3	0.23 ± 0.01	77%

^a^Measurement by dynamic light scattering (25°C, *n* = 3). ^b^Estimated by transmission electron microscope images (*n* = 30). ^c^Measurement by RiboGreen assay.

The pH-responsiveness is a key property for the tumor selective DDS with CB-LNPs. Thus, we evaluated the ζ-potentials of the CB-LNPs obtained at pH 5.5, 6.5, and 7.4 (Figure 2(j)). For the CB20-, CB70-, and CB110-LNPs, the estimated ζ-potentials were + 28.3 ± 1.1, +33.4 ± 0.1 and + 24.4 ± 1.8 mV at pH 5.5, +8.8 ± 0.3, +12.9 ± 0.7 and + 7.4 ± 0.1 mV at pH 6.5, −4.7 ± 0.2, −4.0 ± 0.1 and −6.3 ± 0.5 mV at pH 7.4, respectively, suggesting a relatively large pH-dependency on the surface change. Note that the pH in blood compartment is 7.4 and major serum components are neutral or negatively charged, and the neutral or negative charge is integral to exhibit the antifouling behavior and stealth functionality. Moreover, lower pH values (6.5 and 5.5) mimicked tumor conditions such as the extracellular tumor microenvironment (pH 6.5–6.7) and late endosomal environment (pH 5.5) [36]. Therefore, the above results provide grounds for the expectation that the obtained CB-LNPs can interact appropriately with the target tumor tissues and facilitates endocytosis and endosomal escape. In the case of the control group, *i.e*. DODAP-LNPs, pH-dependent changes were also observed, but were limited only in positive area (+ 17.0 ± 2.9 mV at pH 5.5, + 4.4 ± 0.6 mV at pH 6.5, and + 12.4 ± 1.6 mV at pH 7.4). Regarding this point, we used DODAP as an ionizable cationic lipid and dimethylamine group equipped their weak pH-responsiveness, this characteristic was influenced by the pH-responsiveness of PEG-LNPs (+ 17.0 ± 2.9 mV at pH 5.5, +1.9 ± 0.3 mV at pH 6.5, and −0.2 ± 0.4 mV at pH 7.4). However, the degree and sensitivity of pH-responsiveness were weaker than that of the CB-LNPs.

### 3.2. Cellular uptake and endosomal escape study

**Figure 3.**
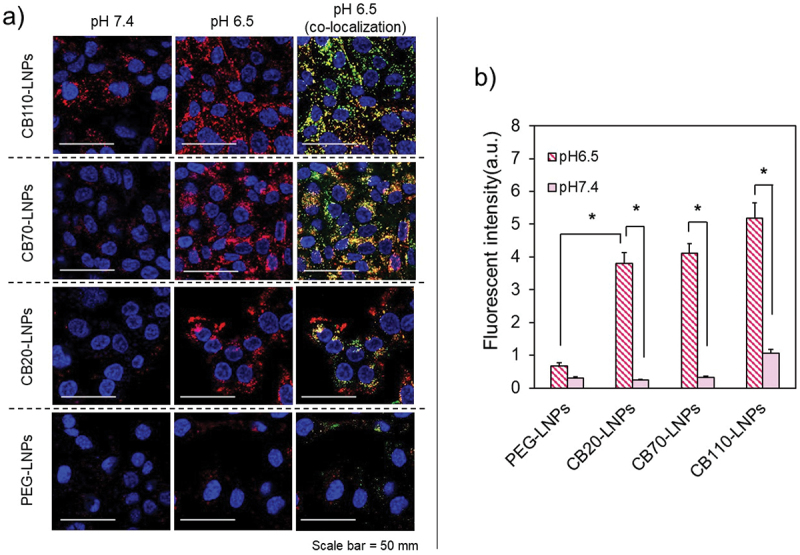
Confocal laser scanning microscopy (CLSM) observation of pH-dependent cellular uptake. (a) SK-OV-3-luc cells were incubated with Alexa Flour 647-siRNA conjugated lipid nanoparticles (LNPs) (red) for 72 h, and then stained for early endosome (green) and nucleus (blue). Colocalization is shown in yellow. Scale bar = 50 µm. (b) Quantitative analysis of cellular uptake. Statistical significance was evaluated using Student’s *t*-test, *p < 0.05. The results are expressed as means ± standard deviation (*n* = 30).

**Figure 4.**
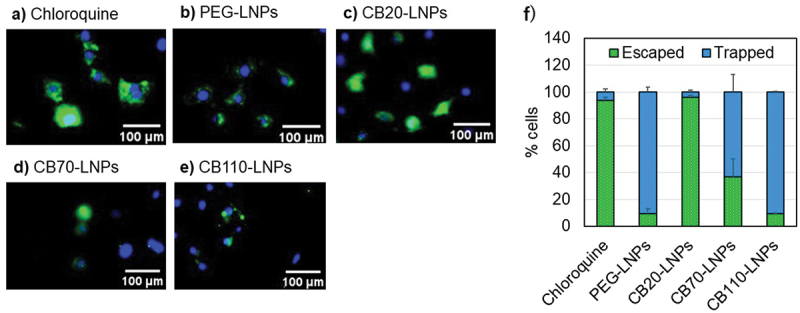
Endosomal escape analysis of LNPs in SK-OV-3-luc cells cultured at pH 6.5. (a-e) Cytosolic distribution of calcein after 2 h co-incubation of each LNPs and calcein. Cytosolic localization of calcein (green). Nuclei were stained using Hoechst 33,342 (blue). Endosomal escape is indicated by diffuse fluorescence throughout the cell, whereas punctate fluorescence represents endosomal trapping. (f) Percentage of cells showing endosomal escape (diffused fluorescence) and endosomal trapping (punctate fluorescence) as quantified using BZ-X800 Analyzer (ver. 1.1.24) (*n* = 30 cells). Scale bar (White) = 100 µm. The results are shown as mean ± SD.

To evaluate the effects of the DP values in PGlu(DET-Car) *in vitro*, we prepared fluorescent-labeled CB-LNPs with siRNA-Alexa Flour 647 conjugates, and the cellular uptake abilities were investigated against human ovarian cancer SK-OV-3-luc cells. As shown in Figure 3(a), the cellular uptake behaviors were fundamentally affected by PGlu(DET-Car) and the DP values. CB110-LNPs (red) demonstrated better internalization at pH 7.4 and 6.5 against SK-OV-3-luc cells than CB20-LNPs. CB70-LNPs and CB20-LNPs showed comparable cellular uptakes at both pH 7.4 and 6.5. PEG-LNPs showed slight cellular uptake under this experimental condition. Additionally, the fluorescent signals from CB110-, CB70-, and CB20-LNPs (red) were co-localized with lysosomes (green) at pH 6.5 (Figure 3(a)), suggesting potential endocytosis-mediated cellular uptake. The quantitative data analysis clearly demonstrates the advantage of the utilization of PGlu(DET-Car). Compared to PEG-LNPs, the fluorescence intensities of pH-responsive CB20-LNPs were significantly higher at pH 6.5, and this tendency was further enhanced with CB70- and CB110-LNPs (Figure 3(b)). Although the above data indicated the utility of PGlu(DET-Car) for inducing the endocytosis-mediated cellular uptake, further evidence is needed to fully clarify the existence of red signals, *i.e*. LNPs, in cytoplasm. Thus, we investigated the endosomal escape ability of CB-LNPs using calcein assay method [37,38]. Calcein is a s cell membrane impermeable dye, and they can internalize into the cells through co-endocytosis with LNPs. When LNPs interact with endosome membrane followed by endosomal rupture, the calcein is released into the cytosol, and then the diffused fluorescence signals can be observed in the cell. While the punctate fluorescence is entrapped within endosomes. CB-LNPs treated group showed the DP dependent-increment of fluorescence signals (Figure 4), suggesting the lower DP induced enhanced endosomal escape. The CB20-LNPs showed the comparable ability of endosomal escape with that of chloroquine treated group (positive control) (Figure 4(f)). On the other hand, PEG-LNPs exhibited only punctate fluorescence, probably due to the PEG dilemma, indicating the importance of pH-responsiveness and number of betaine units in PGlu(DET-Car) on the cellular uptake and endosomal escape of CB-LNPs.

### 3.3. Membrane fusion assay

**Figure 5.**
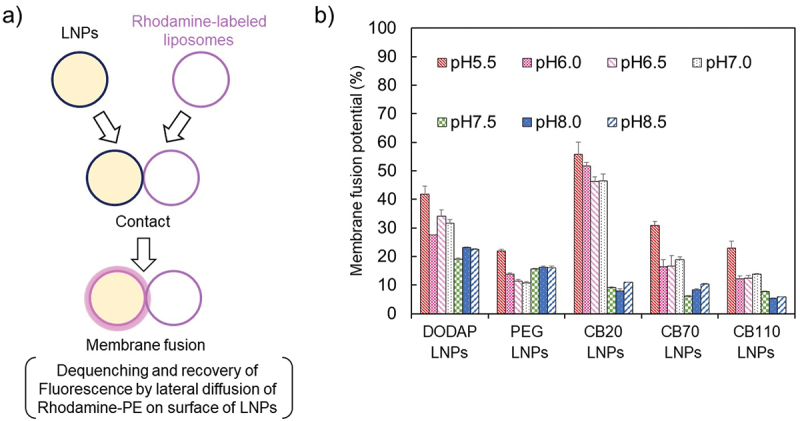
Membrane fusion assay. (a) Schematic of membrane fusion assay. (b) pH-dependent potential of membrane fusion (*n* = 3).

Interactive property of the LNP surface is vital to facilitate endocytosis and endosomal escape to deliver oligonucleotide therapeutics, and efficiency of membrane fusion may be an indicator of gene repression efficiency [28,29]. In this regard, the membrane fusion assay is useful to evaluate the functionality of the membrane using fluorescent lipids containing liposomes as a mimic of endosomal membranes. As shown in Figure 5(a), we initially prepared the fluorescent quenched liposomes and co-incubated them with each LNP sample. When the fluorescent quenched liposomes fused with LNPs, a part of the fluorescent lipids in the liposomes were transferred into the LNP membrane, and a detectable fluorescent signal was generated. In the present study, CB-LNPs were co-incubated with liposomes containing 10% Rhodamine-PE. After 1 h co-incubation, the CB20-LNPs showed clear pH-responsiveness and represented the highest membrane fusion potential compared with other LNPs (Figure 5(b)). PEG-LNPs exhibited low potential across the pH range, thereby reproducing PEG dilemma-like difficulty of membrane fusion in our experimental system. It is worth noting that CB70- and 110-LNPs which exhibited better cellular uptake performances. (Figure 3(b)) showed the lower membrane fusion potential than that of DODAP- and CB20-LNPs, indicating the importance of the balance between PGlu(DET-Car) length and membrane fusion ability.

### 3.4. Gene silencing assay

**Figure 6.**
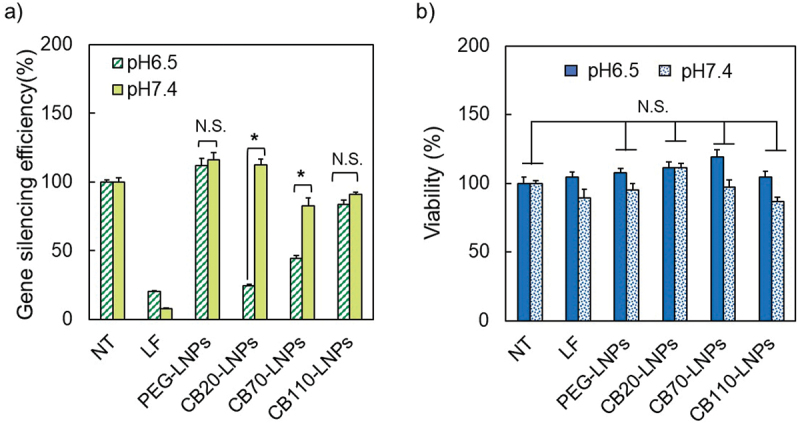
Effect of pH on (a) gene silencing and (b) cell viability with PGlu(DET-Car)-coated lipid nanoparticles (LNPs) against SK-OV-3-luc cells. NT: Non-treatment, LF: Lipofectamine RNAi MAX. (*n* = 3–6). Statistical significance was evaluated using Student’s *t*-test, *p < 0.05. The results are expressed as means ± standard deviation.

We evaluated the *in vitro* gene suppression by measuring the silencing efficiency of luciferase activity (Figure 6(a)). siLuc-encapsulated LNPs were co-incubated for 72 h with SK-OV-3-luc. We found that CB20-LNPs induced a pH-responsive gene suppression against both cell lines, and the efficiency was comparable to that of the lipofectamine positive control which does not show the pH-responsiveness. CB70-LNPs also showed the silencing efficiency, but the degree of pH-responsiveness was relatively weaker than that of CB20-LNPs. While PEG- and CB110-LNPs showed almost no gene suppression effect at both pH conditions. Additionally, cytotoxicity was evaluated by the CCK-8 assay. We confirmed that there was no pH-dependent reduction in the cell viability (Figure 6b). These results indicate the utility of shorter PGlu(DET-Car) as a characteristic of LNPs, and we show that precise tuning of the DP values in the PGlu(DET-Car) segment is crucial to maximize the effects of pH-responsive drug delivery.

## 4. Discussion

PEGylated LNPs have attracted increasing attention as carriers of nucleic acids; however, the insufficient cellular uptake and endosomal escape are often encountered as a result of the surface modification of drug carriers using PEG [15,16]. Some reports have indicated that the degree of above insufficiency was affected by the difference in the amount of PEG-lipid conjugates or the molecular weight around single carrier [39,40]. Thus, the fine tuning of the polymer components is an important issue for carrier design and functionality. To overcome this PEG-related insufficiency, we developed a pH-responsive synthetic polymer, PGlu(DET-Car), and demonstrated its utilities through PGlu(DET-Car) coated quantum dots [17], gold nanoparticles [18], siRNA-loaded LNPs [19], and plasmid DNA-loaded polyion complex [20]. However, the effect of the molecular length of the PGlu(DET-Car) chain remains to be clarified yet. Herein, we prepared three distinct CB-LNPs with different PGlu (DET-Car) lengths, *i.e*. CB20-, CB70-, and CB110-LNPs, using a microfluidic device. We evaluated their potentials in comparison with the PEG-LNP, which were coated with comparable repeating units of ethylene glycol (DP = ca. 110). The obtained CB-LNPs exhibited clear pH-responsiveness, especially with the negative charges at pH7.4 for the expected presence of antifouling ability in the blood constituent. Between pH 6.5–7.4, CB20-, CB70-, and CB110-LNPs showed proactive cellular uptake likely due to their surface charge. Note that CB20-LNPs showed a clear difference in the results of membrane fusion assay between pH 7.0 and pH 7.5, thus their cellular uptake at an acidic pH could be induced by strong electrostatic interactions which are evident in their ζ potential values at same pH condition (pH = 6.5). Additionally, this altering of the membrane fusion potential is crucial to accelerate endosome escape of CB20-LNPs, resulting in effective and pH-selective gene silencing. Although CB110-LNPs showed better cellular uptake at pH 6.5, the *in vitro* gene suppression was negligible, because CB110-LNPs did not show pH-dependent membrane fusion potential and the results were comparable to that of PEG-LNPs. These results indicate the importance of surface density to facilitate the membrane fusion. A similar trend was observed when the surface of liposome was highly modified with membrane fusion proteins, the membrane fusion process was gradually restrained in response to its surface density [41]. Although further research is required to reveal the complete relations, the net charge in CB110-LNPs is highly cationic and interacted strongly with the anionic liposome membrane, resulting in the interception of the membrane fusion process. The above results now raised another question regarding the influence for phase transition ability of the DSPE-PGlu(DET-Car). It is well-known that the lamellar-to-hexagonal II phase transitions are fundamental driving force for membrane fusion process. Given all components including DODAP, DOPE and DSPE-PGlu(DET-Car) would interact each other and work for the phase transition, the molecular weight of polymer components might affect those process. In this sense, our results also support one possibility that the high molecular weight of PGlu(DET-Car) prohibited the lipid-lipid interactions, probably because of the steric hindrance, resulting in inefficient membrane fusion [8,42].

In summary, we identified the importance of design of polymers and their molecular weight in the LNP system that can deliver siRNA to cancer cells. PGlu(DET-Car) modification around the LNP surface induced pH-responsive acceleration of cellular uptake, but tuning of the DP value and cationic charge were required to obtain high membrane fusion potential, endosome escape, and better gene silencing effects. These results demonstrate that the appropriate modification of PGlu(DET-Car) can be used to construct the next generation of drug carrier systems which have the potential to become a platform for future tumor treatment.

## References

[1] Kulkarni JA, Witzigmann D, Thomson SB, et al. The current landscape of nucleic acid therapeutics. Nat Nanotech. 2021;16(6):630–12. doi: 10.1038/s41565-021-00898-0 34059811

[2] Hu B, Weng Y, Xia XH, et al. Clinical advances of siRNA therapeutics. J Gene Med. 2019;21(7):e3097. doi: 10.1002/jgm.3097 31069898

[3] Mitchell MJ, Billingsley MM, Haley RM, et al. Engineering precision nanoparticles for drug delivery. Nat Rev Drug Discov. 2020;20(2):101–124. 33277608 10.1038/s41573-020-0090-8 PMC7717100

[4] Urits I, Swanson D, Swett MC, et al. A review of Patisiran (ONPATTRO®) for the treatment of polyneuropathy in people with hereditary transthyretin amyloidosis. Neurol Ther. 2020;9(2):301–315. 32785879 10.1007/s40120-020-00208-1 PMC7606409

[5] Wang X. Safety and efficacy of the BNT162b2 mRNA Covid-19 vaccine. N Engl J Med. 2021;383:2603–2615. doi: 10.1056/NEJMc2036242 PMC7745181 33301246

[6] Hassett KJ, Benenato KE, Jacquinet E, et al. Optimization of lipid nanoparticles for intramuscular administration of mRNA vaccines, Mol. Ther. Mol Ther Nucleic Acids. 2019;15:1–11. doi: 10.1016/j.omtn.2019.01.013 30785039 PMC6383180

[7] Maier MA, Jayaraman M, Matsuda S, et al. A. Akinc, Biodegradable lipids enabling rapidly eliminated lipid nanoparticles for systemic delivery of RNAi therapeutics. Mol Ther. 2013;21(8):1570–1578. doi: 10.1038/mt.2013.124 23799535 PMC3734658

[8] Semple SC, Akinc A, Chen J, et al. Rational design of cationic lipids for siRNA delivery. Nat Biotechnol. 2010;28(2):172–176. doi: 10.1038/nbt.1602 20081866

[9] Du Z, Munye MM, Tagalakis AD, et al. The role of the helper lipid on the DNA transfection efficiency of lipopolyplex formulations. Sci Rep. 2014;4(1):1–6. doi: 10.1038/srep07107 PMC4236742 25407686

[10] Kulkarni JA, Witzigmann D, Leung J, et al. On the role of helper lipids in lipid nanoparticle formulations of siRNA. Nanoscale. 2019;11(45):21733–21739. doi: 10.1039/c9nr09347h 31713568

[11] Lee JB, Zhang K, Tam YYC, et al. A glu-urea-lys ligand-conjugated lipid nanoparticle/siRNA system inhibits androgen receptor expression in vivo. Mol Ther Nucleic Acids. 2016;5(8):e348. doi: 10.1038/mtna.2016.43 28131285 PMC5024509

[12] Qin J, Xue L, Gong N, et al. RGD peptide-based lipids for targeted mRNA delivery and gene editing applications. RSC Adv. 2022;12(39):25397–25404. doi: 10.1039/d2ra02771b 36199352 PMC9450108

[13] Kara G, Calin GA, Ozpolat B. RNAi-based therapeutics and tumor targeted delivery in cancer. Adv Drug Deliv Rev. 2022;182:114113. doi: 10.1016/j.addr.2022.114113 35063535

[14] Liu D, Gao S, Zhai Y, et al. Research progress of tumor targeted drug delivery based on PD-1/PD-L1. Int J Pharm. 2022;616:121527. doi: 10.1016/j.ijpharm.2022.121527 35104594

[15] Hatakeyama H, Akita H, Harashima H. A multifunctional envelope type nano device (MEND) for gene delivery to tumours based on the EPR effect: a strategy for overcoming the PEG dilemma. Adv Drug Deliv Rev. 2011;63(3):152–160. doi: 10.1016/j.addr.2010.09.001 20840859

[16] Zalba S, Ten Hagen TLM, Burgui C, et al. Stealth nanoparticles in oncology: facing the PEG dilemma. J Control Release. 2022;351:22–36. doi: 10.1016/j.jconrel.2022.09.002 36087801

[17] Ranneh AH, Takemoto H, Sakuma S, et al. An ethylenediamine-based switch to render the polyzwitterion cationic at tumorous pH for effective tumor accumulation of coated nanomaterials. Angew Chem Int Ed Engl. 2018;57(18):5057–5061. doi: 10.1002/anie.201801641 29512262

[18] Awaad A, Takemoto H, Iizuka M, et al. Changeable net charge on nanoparticles facilitates intratumor accumulation and penetration. J Control Release. 2022;346:392–404. doi: 10.1016/j.jconrel.2022.04.025 35461967

[19] Sung YJ, Guo H, Ghasemizadeh A, et al. Cancerous pH-responsive polycarboxybetaine-coated lipid nanoparticle for smart delivery of siRNA against subcutaneous tumor model in mice. Cancer Sci. 2022;113(12):4339–4349. 36047963 10.1111/cas.15554 PMC9746038

[20] Shen X, Dirisala A, Toyoda M, et al. pH-Responsive polyzwitterion covered nanocarriers for DNA delivery. J Control Release. 2023;360:928–939. doi: 10.1016/j.jconrel.2023.07.038 37495117

[21] Rajesh M, Sen J, Srujan M, et al. Dramatic influence of the orientation of linker between hydrophilic and hydrophobic lipid moiety in liposomal gene delivery. J Am Chem Soc. 2007;129(37):11408–11420. doi: 10.1021/ja0704683 17718562

[22] Stamatatos L, Leventis R, Zuckermann MJ. Interactions of cationic lipid vesicles with negatively charged phospholipid vesicles and biological membranes. Biochemistry. 1988;27(11):3917–3925. doi: 10.1021/bi00411a005 3415963

[23] Yang Z, Gou L, Chen S, et al. Membrane fusion involved in neurotransmission: glimpse from electron microscope and molecular simulation. Front Mol Neurosci. 2017;10:168. doi: 10.3389/fnmol.2017.00168 28638320 PMC5461332

[24] Zhou P, Bacaj T, Yang X, et al. Lipid-anchored SNAREs lacking transmembrane regions fully support membrane fusion during neurotransmitter release. Neuron. 2013;80(2):470–483. doi: 10.1016/j.neuron.2013.09.010 24120845 PMC3872166

[25] Eckert DM, Kim PS. Mechanisms of viral membrane fusion and its inhibition. Annu Rev Biochem. 2001;70(1):777–810. doi: 10.1146/annurev.biochem.70.1.777 11395423

[26] Harrison SC. Viral membrane fusion. Nat Struct Mol Biol. 2008;15(7):690–698. doi: 10.1038/nsmb.1456 18596815 PMC2517140

[27] Jahn R. Principles of exocytosis and membrane fusion. Ann N Y Acad Sci. 2004;1014(1):170–178. doi: 10.1196/annals.1294.018 15153432

[28] Kong H, Yi K, Zheng C, et al. Membrane-fusogenic biomimetic particles: a new bioengineering tool learned from nature. J Mater Chem B Mater Biol Med. 2022;10(36):6841–6858. doi: 10.1039/d2tb00632d 35781483

[29] Sato Y, Hatakeyama H, Hyodo M, et al. Relationship between the physicochemical properties of lipid nanoparticles and the quality of siRNA delivery to liver cells. Mol Ther. 2016;24(4):788–795. 26678452 10.1038/mt.2015.222 PMC4886930

[30] Belliveau NM, Huft J, Lin PJ, et al. Microfluidic synthesis of highly potent limit-size lipid nanoparticles for in vivo delivery of siRNA. Mol Ther Nucleic Acids. 2012;1:e37. doi: 10.1038/mtna.2012.28 23344179 PMC3442367

[31] Jones LJ, Yue ST, Cheung CY, et al. RNA quantitation by fluorescence-based solution assay: RiboGreen reagent characterization. Anal Biochem. 1998;265(2):368–374. doi: 10.1006/abio.1998.2914 9882416

[32] Pires P, Simões S, Nir S, et al. Interaction of cationic liposomes and their DNA complexes with monocytic leukemia cells. Biochim Biophys Acta Biomembr. 1999;1418(1):71–84. doi: 10.1016/S0005-2736(99)00023-1 10209212

[33] Obata Y, Saito S, Takeda N, et al. Plasmid DNA-encapsulating liposomes: effect of a spacer between the cationic head group and hydrophobic moieties of the lipids on gene expression efficiency. Biochim Biophys Acta. 2009;1788(5):1148–1158. doi: 10.1016/j.bbamem.2009.02.014 19249283

[34] Tanaka H, Takata N, Sakurai Y, et al. Delivery of oligonucleotides using a self-degradable lipid-like material. Pharmaceutics. 2021;13(4):544. doi: 10.3390/pharmaceutics13040544 33924589 PMC8070490

[35] Maeki M, Kimura N, Sato Y, et al. Advances in microfluidics for lipid nanoparticles and extracellular vesicles and applications in drug delivery systems. Adv Drug Deliv Rev. 2018;128:84–100. doi: 10.1016/j.addr.2018.03.008 29567396

[36] Sonawane ND, Thiagarajah JR, Verkman AS. Chloride concentration in endosomes measured using a ratioable fluorescent Cl− indicator: evidence for chloride accumulation during acidification. J Biol Chem. 2002;277(7):5506–5513. doi: 10.1074/jbc.M110818200 11741919

[37] Hausig-Punke F, Richter F, Hoernke M, et al. Tracking the endosomal escape: A closer look at calcein and related reporters. Macromol Biosci. 2022;22(10):e2200167. 35933579 10.1002/mabi.202200167

[38] Liu Y, Liu J. Leakage and rupture of lipid membranes by charged polymers and nanoparticles. Langmuir. 2020;36(3):810–818. doi: 10.1021/acs.langmuir.9b03301 31910024

[39] Sato Y, Note Y, Maeki M. Elucidation of the physicochemical properties and potency of siRNA-loaded small-sized lipid nanoparticles for siRNA delivery. J Control Release. 2016;229:48–57. doi: 10.1016/j.jconrel.2016.03.019 26995758

[40] Yang Q, Jones SW, Parker CL, et al. Evading immune cell uptake and clearance requires PEG grafting at densities substantially exceeding the minimum for brush conformation. Mol Pharm. 2014;11(4):1250–1258. 24521246 10.1021/mp400703d

[41] Xu H, Cai M, Gao J, et al. Membrane protein density determining membrane fusion revealed by dynamic fluorescence imaging. Talanta. 2021;226:122091. doi: 10.1016/j.talanta.2021.122091 33676648

[42] Heyes J, Palmer L, Bremner K, et al. Cationic lipid saturation influences intracellular delivery of encapsulated nucleic acids. J Control Release. 2005;107(2):276–287. doi: 10.1016/j.jconrel.2005.06.014 16054724

